# Prospective, non-blinded, randomized controlled trial on early administration of pulmonary surfactant guided by lung ultrasound scores in very preterm infants: study protocol

**DOI:** 10.3389/fped.2024.1411068

**Published:** 2024-07-10

**Authors:** Jinghui Zhang, Huiqiang Liu, Yahui Zhang, Weiwei Zhu, Yunfeng Liu, Tongyan Han

**Affiliations:** Department of Pediatrics, Peking University Third Hospital, Beijing, China

**Keywords:** lung ultrasonography, scoring, pulmonary surfactant, respiratory distress syndrome, very preterm infants

## Abstract

**Background:**

Bedside lung ultrasonography has been widely used in neonatal intensive care units (NICUs). Lung ultrasound scores (LUS) may predict the need for pulmonary surfactant (PS) application. PS replacement therapy is the key intervention for managing moderate to severe neonatal respiratory distress syndrome (NRDS), with early PS administration playing a positive role in improving patient outcomes. Lung ultrasonography aids in the prompt diagnosis of NRDS, while LUS offers a semi-quantitative assessment of lung health. However, the specific methodologies for utilizing LUS in clinical practice remain controversial. This study hypothesizes that, in very preterm infants [<32 weeks gestational age (GA)] exhibiting respiratory distress symptoms, determining PS application through early postnatal LUS combined with clinical indicators, as opposed to relying solely on clinical signs and chest x-rays, can lead to more timely PS administration, reduce mechanical ventilation duration, improve patient outcomes, and lower the occurrence of bronchopulmonary dysplasia (BPD).

**Methods and design:**

This is a protocol for a prospective, non-blinded, randomized controlled trial that will be conducted in the NICU of a hospital in China. Eligible participants will include very preterm infants (< 32 weeks GA) exhibiting signs of respiratory distress. Infants will be randomly assigned in a 1:1 ratio to either the ultrasound or control group. In the ultrasonography group, the decision regarding PS administration will be based on a combination of lung ultrasonography and clinical manifestations, whereas in the control group, it will be determined solely by clinical signs and chest x-rays. The primary outcome measure will be the mechanical ventilation duration. Statistical analysis will employ independent sample *t*-tests with a significance level set at α = 0.05 and a power of 80%. The study requires 30 infants per group (in total 60 infants).

**Results:**

This study aims to demonstrate that determining PS application based on a combination of LUS and clinical indicators is superior to traditional approaches.

**Conclusions:**

This approach may enhance the accuracy of NRDS diagnosis and facilitate early prediction of PS requirements, thereby reducing the duration of mechanical ventilation. The findings of this research may contribute valuable insights into the use of LUS to guide PS administration.

## 1 Background

Neonatal respiratory distress syndrome (NRDS), resulting from a deficiency in pulmonary surfactant (PS), stands as a primary cause of mortality among premature infants. Currently, clinical symptomatology, combined with chest x-rays, constitute the primary diagnostic modalities for NRDS. However, the subjective nature of symptom recognition and the limitations of chest x-rays, including poor sensitivity and specificity along with radiation exposure, pose challenges for clinicians in the early diagnosis of NRDS. In recent years, bedside lung ultrasonography has gained widespread utilization in neonatal intensive care unit (NICU) settings, with numerous studies showcasing its substantial advantages in NRDS diagnosis (1). Consequently, the latest guidelines have recommended the adjunctive use of bedside lung ultrasonography in NRDS diagnosis (2).

Continuous positive airway pressure ventilation and surfactant replacement therapy stand as key interventions for moderate to severe NRDS (3). Early administration of PS has demonstrated positive roles in reducing mortality, improving prognosis, shortening the duration of invasive ventilation, and mitigating the occurrence of bronchopulmonary dysplasia (BPD) among premature infants (4, 5). A study suggests that prophylactic PS administration within 30 min postpartum significantly diminishes the severity and incidence of respiratory distress syndrome (RDS), thereby reducing neonatal mortality and pneumothorax risks (6). Additionally, the 2022 Update of the European Consensus Guidelines on the Management of Respiratory Distress Syndrome strongly recommends early initiation of PS therapy in symptomatic patients with NRDS prior to clinical deterioration, advocating for less-invasive delivery methods (2). Nevertheless, in clinical practice, not all premature infants presenting with respiratory distress symptoms manifest NRDS. Transient RDS, neonatal pneumonia, and meconium aspiration syndrome can also precipitate early respiratory distress. Autopsy findings indicate misdiagnosis rates of NRDS as high as 57% in premature infants (7). For mild NRDS cases, relief through continuous positive airway pressure ventilation alone may suffice, obviating potential airway injury from PS administration. Prompt diagnosis of etiology and decision-making regarding PS administration in the early postnatal period poses a dilemma for clinicians. Current guidelines recommend early PS therapy when infants require respiratory support with positive end-expiratory pressure (PEEP) exceeding 6 cmH2O and fraction of inspired oxygen (FiO_2_) surpassing 0.3; however, there is no consensus on the criterion and cut-off to adopt for surfactant treatment (2, 8). However, patient FiO_2_ requirements vary vastly with postnatal time and respiratory support methods. Moreover, subjective symptom recognition and severity assessment lack objective criteria, further complicating the early decision-making process regarding PS application. On the one hand, waiting to fulfill FiO_2_ criteria for surfactant replacement therapy can lead to delayed administration and reduce the potential beneficial effects of the surfactant (9). On the other hand, loosening the criteria for medication use may result in excessive application of PS. Thus, balancing timely treatment initiation against the risk of overuse remains a challenge (10).

Lung ultrasound scores (LUS) provide a quantitative evaluation of lung ultrasound results.

Numerous studies have shown that LUS can assist in assessing the need for PS application in patients with NRDS. In a prospective study from 2018 involving 133 premature infants born at a gestational age (GA) of less than 30 weeks, it was confirmed that LUS correlated significantly with oxygen saturation and FiO_2_ during the early postnatal period. This correlation helps determine the respiratory status in infants’ and can predict the necessity for PS administration, with the likelihood of PS administration increasing from 51% to 82% or 92% when LUS are more than 6 out of 8 (11). Alternatively, LUS exhibited the highest predictive ability for the initial PS administration compared with FiO_2_ and Silverman scores (12). Additionally, retrospective studies have indicated that utilizing LUS to anticipate PS administration can reduce the misdiagnosis rate of RDS and subsequently lower PS usage by 30.1% (13). Furthermore, LUS offer significant advantages in promptly determining the need for PS application in infants and facilitating early PS administration, within 3 h postnatally. Research has shown that LUS can shorten the durations of both invasive and non-invasive ventilation, thereby reducing the incidence of subsequent BPD (14).

In existing studies, the utilization of LUS to assess NRDS often involves simplified adaptations from adult LUS systems. A unified standard for LUS assessment in neonates remains elusive (14, 15). Variations exist in the scoring locations and methods of LUS applied across different studies. Furthermore, a consensus on the threshold of LUS for PS application has yet to be established, hindering its widespread clinical application. Therefore, this study aimed to demonstrate, through a randomized controlled trial, that for very preterm infants (< 32 weeks GA) experiencing respiratory distress, determining PS application based on LUS combined with clinical indications can lead to more accurate NRDS diagnosis compared with traditional methods that rely on clinical indications and chest x-rays. Hence, this approach may facilitate the early and appropriate use of PS, within 3 h postnatally, thereby reducing mechanical ventilation duration, improving patient outcomes, lowering the incidence of BPD, and decreasing medical expenses. Furthermore, lung ultrasonography can partially substitute for chest x-rays in the diagnosis of early NRDS, significantly reducing early radiation exposure for patients.

## 2 Methods

### 2.1 Study design

**Figure 1:**
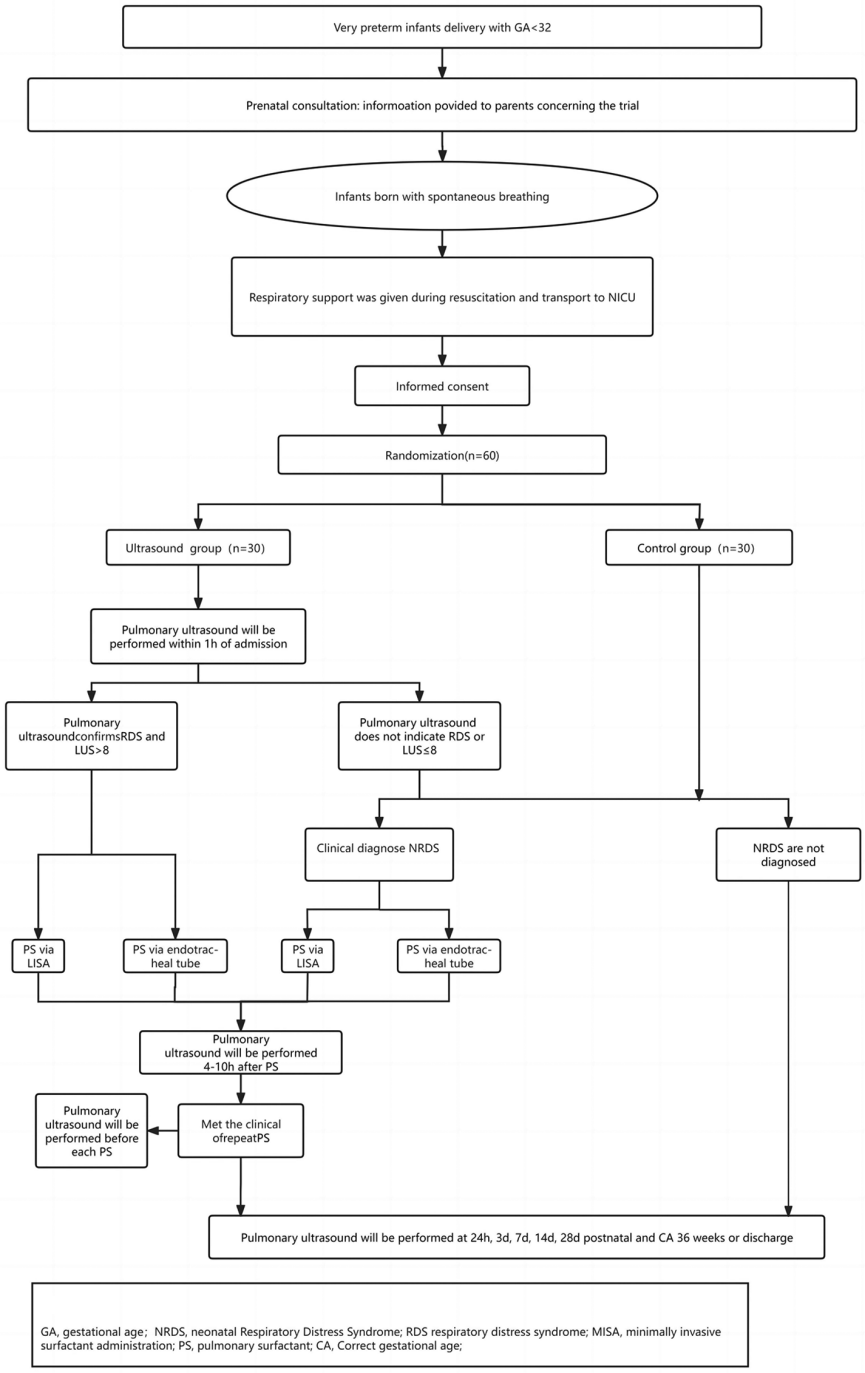
Fowchart of participant selection and treatment.

This prospective, non-blinded, randomized controlled trial is to be conducted in the NICU of a tertiary referral hospital in China. This NICU serves as the regional referral center for high-risk newborns and pregnant women, with an annual delivery volume exceeding 6,000, including approximately 200 very preterm infants (< 32 weeks GA). This study primarily targeted very preterm infants presenting with symptoms of respiratory distress. Following enrollment, participants will undergo standardized trial procedures (Figure 1).

All very preterm infants will undergo antenatal consultations before birth. Neonatologists will engage in thorough discussions with the parents of premature infants, informing them about the risks associated with premature birth at this GA and outlining the treatment process post-birth. Before delivery, a neonatal resuscitation team comprising neonatal and obstetric specialists will be established. Following delivery, premature infants will be transferred within the hospital using transport incubators, and a T-piece resuscitator will be utilized during the transfer process. Within 1 h after birth, all enrolled infants will undergo lung ultrasonography, and those meeting the diagnostic criteria will receive PS as per the trial protocol. Subsequently, they will undergo regular lung ultrasound monitoring until discharge or at 36 weeks corrected age (CA).

Centers participating in the study routinely use lung ultrasonography. Two ultrasound physicians and five neonatologists will participate in this study. Both ultrasound physicians have received comprehensive training in lung ultrasonography and LUS application. Lung ultrasonography will be performed by two ultrasound physicians, who each have more than 2 years of experience in neonatal lung ultrasound and have received systematic training before the study, including study protocols, lung ultrasound scoring, and interpretation of lung ultrasound images. In order to ensure the consistency of scoring results, the two doctors will respectively determine the LUS based on ultrasound images. If the scores are consistent, the results will be recorded. If the score difference is ≥2, the final score will be determined by the two doctors after discussion.

Other research personnel have completed standardized training, including familiarization with the trial protocol, methods of recording case records, and criteria for assessing complications.

Lung ultrasonography will be conducted using the Mindray M9 ultrasound system equipped with a linear, high frequency (10–15 MHz) transducer.

### 2.2 Participants

#### 2.2.1 Inclusion criteria

Infants who meet all of the following criteria will be included**:** (1) very preterm infants with a GA of less than 32 weeks; (2) presence of respiratory distress after birth, such as a respiratory rate of more than 60 breaths/min, grunting, nasal flaring, intercostal retractions, and/or cyanosis; (3) born in the authors’ hospital.

#### 2.2.2 Exclusion criteria

Infants who meet any of the following criteria will be excluded: (1) infants who have received PS treatment in the delivery room or external setting after birth; (2) infants with known or confirmed congenital abnormalities, especially cardiopulmonary deformities during diagnosis or treatment; (3) presence of severe complications at birth (severe asphyxia, hemorrhagic shock, pneumonia, pneumothorax, early-onset sepsis, and pleural effusion) or other diseases not caused by PS deficiency; (4) infants who die within 72 h after birth, are transferred to another hospital for surgery, or have incomplete data; and (5) those with families who do not consent to participate in this study.

### 2.3 Randomization

Following transfer to the NICU ward, eligible patients will be randomly assigned to two groups (ultrasound group, control group) in a 1:1 ratio. Randomization will be conducted using a predetermined random number table generated by an independent statistician using computer software (R software) and concealed within sealed opaque envelopes. For multiple births, randomization will be performed individually.

### 2.4 Quality control

This trial adheres to the requirements of the Ethics Committee and will be conducted under the supervision of the Ethics Committee of the participating institution. Researchers are obligated to diligently complete the case report forms with accuracy, detail, and honesty, ensuring the authenticity and reliability of the recorded information. All observations and findings during clinical validation will be verified to guarantee data reliability, and all conclusions drawn from clinical validation will be rooted in original data. Appropriate data management measures will be implemented throughout clinical validation and data processing. Data entry into the SPSS version 20.0 software (Armonk, NY: IBM Corp) from the case report forms will be double-checked by two individuals.

### 2.5 Blinding

In this study, as the administration of the first dose of PS depends on the initial lung ultrasound results, ultrasound physicians and neonatologists will be informed of the infant's group assignment at the beginning. However, subsequent ultrasound results after the first dose will not be disclosed to the clinical team to prevent any influence on the assessment of indications for the second dose of PS. In addition, blinding will be implemented for the members involved in outcome assessment and data analysis to reduce bias.

### 2.6 Assessment points

The main assessment and recording of the infants’ indicators will occur at the following time points (Table 1).

**Table 1:** Neonatal assessment chart.

Measure	Assessment point							
	Allocation within 1 h after birth	4–10 h after PS	24 h	3 days	7 days	14 days	28 days	36 weeks CA/discharge
LUS	×	×	×	×	×	×	×	×
Clinical index								
Silverman Anderson score	×	×	×	×	×	×	×	×
Respiratory support mode	×	×	×	×	×	×	×	×
Ventilator parameters	×	×	×	×	×	×	×	×
Medication use of PS	×		×					
Outcomes assessment								
NIV (hours)								×
IMV (hours)								×
Supplemental oxygen (days)								×
PS (%)								×
Early PS (%)								×
Birth to the application of 1st PS (min)								×
LOS								×
Costs associated with hospital care								×
Complications								
hsPDA (%)								×
BPD (%)								×
IVH (%)								×
NEC stage I or II (%)								×
ROP (%)								×

NIV, non-invasive ventilation; IMV, invasive mechanical ventilation; Early PS, pulmonary surfactant administered within 3 h after birth; LOS, length of stay; hsPDA, hemodynamically significant patent ductus arteriosus; IVH, intraventricular hemorrhages; BPD, bronchopulmonary dysplasia; NEC, necrotizing enterocolitis; ROP, retinopathy of prematurity.

### 2.7 Planned intervention

All study participants will receive initial resuscitation in the delivery room or operating room following the latest European Resuscitation Council Guidelines for newborns (16), which dictate whether non-invasive or invasive ventilation should be administered. Subsequently, they will be transported to the NICU using a transport incubator. Throughout ventilation and transportation, a T-piece resuscitator will be utilized to provide either non-invasive or invasive respiratory support (Initial parameters: PEEP = 6 cmH2O, PIP = 15–20 cmH2O, FiO_2_≤0.4) in order to maintain the patient's target oxygen saturation. If the target oxygen saturation is not achieved, PEEP and FiO_2_ can be upregulated, but in order to avoid pneumothorax, PEEP should be controlled at ≤8 cmH2O (2). Our NICU implements the “golden one-hour” management for premature infants (17, 18). Premature infants with successful recovery will be transferred to the NICU ward within 15 min and reach a relatively stable state shortly after birth. Upon admission to the ward, all preterm infants will be randomly assigned to either the ultrasound or control group.

#### 2.7.1 Ultrasound group

**Figure 2:**
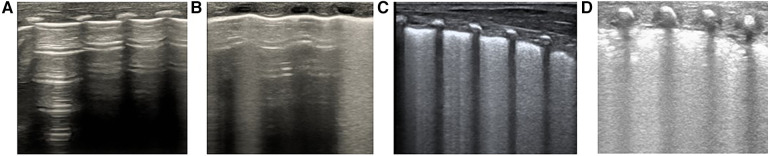
Calculating lung ultrasonography scores. Segmentation: Each lung is divided into 3 regions (anterior L12/R12, lateral L34/R34, and posterior L56/R56), totaling 6 regions. Scoring: The higher the score, the more severe the condition, with a total score of 18 points. The detailed scoring criteria are as follows: (**A**) 0 points indicate normal lung ventilation, characterized by A-lines with sporadic B-lines; (**B**) 1 point signifies moderate reduction in lung ventilation, including fused B-lines and interstitial syndrome (B-line accumulation <50% of the lung field examined); (**C**) 2 points denote severe reduction in lung ventilation, with a small amount of localized subpleural consolidation (B-line accumulation ≥50% of the lung field examined; B-line covers more than half of the intercostal space); (**D**) 3 points represent extensive lung consolidation.

Lung ultrasonography will be performed within 1 h of admission, followed by lung ultrasound scoring (refer to Figure 2 for the segmentation and scoring methods). In the case of different score patterns in the same area, the worst will be selected. If the lung ultrasound confirms RDS and LUS is more than 8, a full dose of PS will be promptly administered at 200 mg/kg (Poractant Alfa, Curosurf®). If the lung ultrasound does not indicate RDS or if LUS is 8 or less, clinical monitoring will continue. Should the clinical criteria for NRDS be met, a full dose of PS will be administered at 200 mg/kg (Poractant Alfa, Curosurf®). In terms of the administration method, less-invasive surfactant administration (LISA) will be employed for infants in a non-invasive ventilation state, while those in an invasive ventilation state will receive PS via endotracheal tube. Lung ultrasound and scoring will be repeated 4–10 h post the first PS dose administration (with scoring results withheld from the clinical team). Repeated PS administration will occur if the criteria are met, with lung ultrasound scoring conducted prior to each administration (with scoring results withheld from the clinical team). Repeated PS administration will be completed via endotracheal tube.

#### 2.7.2 Control group

In cases where clinical diagnosis confirms NRDS, PS will be administered at a dosage of 200 mg/kg (Poractant Alfa, Curosurf®). LISA will be employed for infants in a non-invasive ventilation state, while those in an invasive ventilation state will receive PS via endotracheal tube. If clinical manifestations do not meet the diagnosis of NRDS, PS will not be administered.

All enrolled infants will undergo lung ultrasound scoring at 24 h, 3 days, 7 days, 14 days, 28 days, and 36 weeks CA (prior to discharge) (with scoring results withheld from the clinical team).

### 2.8 Therapy

Lung ultrasound procedure: The lungs will be partitioned based on the six-region method in the pulmonary ultrasound guidelines. Each lung will be divided into three regions: anterior, lateral, and posterior lung area. To do this, the anterior axillary line and the posterior axillary line will be used as boundaries. Both lungs will be divided into a total of six regions (19). An ultrasound probe perpendicular to the intercostals will be used to scan each area from the top to bottom and score it. A score of 0–3 (as proposed by Bratet al.) will be assigned to each scan area based on the ultrasound detected pattern; in the case of different score patterns in the same area, the worst will be selected (Figure 2) (14).

RDS lung ultrasound diagnostic criteria: (1) Lung consolidation with air bronchograms: this is the most crucial ultrasound manifestation of RDS, characterized by: (i) The degree and extent of consolidation correlate with disease severity. Mild RDS may exhibit consolidation limited to the subpleural areas, appearing focal and small in scope, while severe RDS demonstrates expanded consolidation areas, extending into deeper lung fields; (ii) consolidation may be observed in different lung fields on bilateral lungs or restricted to certain intercostal spaces on one side; and (iii) air bronchograms present as dense, snowflake-like, or spot-like patterns, with the consolidation area showing heterogeneous hypoechoic areas that are easily distinguishable from surrounding lung tissue; (2) Abnormal pleural lines and disappearance of A-lines; (3) Non-consolidated areas show changes resembling interstitial syndrome; (4) Bilateral points: these may be present during the acute phase of mild RDS or the recovery phase of severe RDS; and (5) pleural effusion: infants may exhibit varying degrees of unilateral or bilateral pleural effusion (19).

Clinical diagnostic criteria for NRDS: Within 24 h of birth, if an infant experiences respiratory distress, they are supported with nasal intermittent positive pressure ventilation (NIPPV) or nasal continuous positive airway pressure (NCPAP). Diagnosis of NRDS occurs when the infant exhibits progressively worsening symptoms, such as a respiratory rate of more than 60 breaths/min, grunting, nasal flaring, intercostal retractions, and/or cyanosis, along with a peripheral capillary oxygen saturation (SpO2) of 85% or less, a Silverman Anderson Score (SAS) of more than 5, or an hourly increase in SAS by more than 2 while the FiO_2_ is more than 0.4 (or >0.3 for those with a GA<28 weeks). These symptoms may or may not be accompanied by typical chest x-ray findings.

Indications for repeated use of PS include the following: within 72 h after birth (that is, 10–70 h after the initial PS administration), if respiratory distress persists, chest x-ray findings confirm the diagnosis of NRDS, and other causes of respiratory difficulties have been excluded.

Indications for endotracheal intubation and invasive mechanical ventilation within 72 h after birth include: (1) severe respiratory acidosis indicated by blood gas analysis 2 h after the initial PS administration (PaCO2 >65 mmHg, pH <7.20); (2) hypoxemia, with an FiO_2_ of more than 0.4 and an SpO2 of less than 0.85; (3) recurrent episodes of respiratory pauses, more than 3 episodes of spontaneously recoverable respiratory pauses per hour, or one episode of respiratory pause requiring positive pressure ventilation within 24 h; (4) an increase in SAS by more than 2 per hour, or an SAS of more than 5 persisting for more than 2 h; (5) pulmonary hemorrhage; (6) respiratory or cardiac arrest requiring endotracheal intubation for resuscitation; and (7) required by NICU physicians for respiratory support to ensure the infant will receive optimal treatment. Criteria for extubation include an FiO_2_ of less than 0.3 and a mean airway pressure (MAP) of less than 7–8 cmH2O.

Criteria for discontinuation of non-invasive ventilation: (1) more than 72 h after birth; (2) a PEEP of 3–5 cmH2O or less during NCPAP, or an MAP of less than 6–7 cmH2O during NIPPV; (3) an FiO_2_ of 25% or less; (4) an SAS of less than 3; and (5) absence of respiratory pauses or bradycardia requiring stimulation for recovery. If all five conditions are met and sustained for over 24 h, discontinuation of non-invasive ventilation may be considered based on clinical assessment. If any of the following conditions arise after discontinuation of non-invasive ventilation, encompassing: (1) an FiO_2_ of more than 0.25 and an SpO2 of less than 85%; (2) an SAS of more than 3; or (3) three or more episodes of spontaneously recoverable respiratory pauses or bradycardia within 24 h, non-invasive ventilation will be reinstated. Reassessment for discontinuation of non-invasive ventilation will not be conducted until at least 48 h later.

All study participants will receive caffeine citrate within 24 h after birth to prevent respiratory pauses, with an initial loading dose of 20 mg/kg and a maintenance dose of 5–10 mg/kg per day.

### 2.9 Outcome measures

The primary outcome measure for this study is the duration of mechanical ventilation (both invasive and non-invasive) during hospitalization in the ultrasound and control groups. Secondary outcome measures encompass: (1) the utilization rate of PS in both groups; (2) the proportion of infants receiving the first dose of PS early (within 3 h after birth) and the time from birth to the initial PS administration; (3) the oxygen concentration and the worst oxygenation index (OI) at different time points following PS administration; (4) LUS at various intervals following PS administration (at 3 days, 7 days, 14 days, 28 days, and before discharge/at 36 weeks CA; and (5) the incidence rates of major complications in preterm infants such as BPD, intraventricular hemorrhage (IVH), retinopathy of prematurity (ROP), and necrotizing enterocolitis (NEC).

### 2.10 Data collection and diagnoses

Patient demographic data mainly include: (1) GA, birth weight, singleton or multiple births, sex, prenatal steroid use, mode of delivery, presence of intrauterine distress, and maternal pregnancy complications; and (2) Apgar scores at 1 min, 5 min, and 10 min, as well as umbilical cord blood gas analysis.

Clinical information at each time point (at admission, 1 h after birth, 4–10 h after PS administration, 24 h, 72 h, 7 days, 14 days, 28 days, and before discharge or at 36 weeks CA) include (1) timing, dosage, and method of the initial PS administration; (2) SAS, respiratory machine mode, oxygen saturation index, and FiO_2_ levels at each time point; and (3) LUS at each time point. LUS will collect individual scores for each lung field as well as the total score.

Complications and outcomes: (1) Respiratory complications: this includes the occurrences of infectious pneumonia, pneumothorax, pulmonary hemorrhage, and BPD, along with their severity grading; (2) Cardiovascular complications: specifically, hemodynamically significant patent ductus arteriosus (hsPDA); (3) Other complications in premature infants: these encompass IVH, periventricular leukomalacia, NEC, late-onset sepsis, ROP, intrauterine growth restriction, and more; and (4) Length of hospital stay and associated costs.

BPD diagnosis will adhere to the 2018 National Institute of Child Health and Human Development (NICHD) classification. Diagnosis of hsPDA will rely on clinical signs and echocardiography (20). IVH and white matter injury will be diagnosed via cranial ultrasound, with IVH graded using the Papile classification (21). NEC staging will follow modified Bell criteria (22). ROP diagnosis and staging will be based on fundoscopic examinations conducted by ophthalmologists.

### 2.11 Safety procedures

Throughout the study duration, researchers will continuously monitor all enrolled infants to ensure their safety. Any occurrence of severe or significant adverse events will be promptly reported to the principal investigator for assessment. This study strictly adheres to the principles of human subject protection, and in the event of adverse events, regardless of their association with the study, the trial will be promptly terminated.

### 2.12 Sample size calculation

With the duration of mechanical ventilation as the primary outcome measure for both groups, we anticipate that patients in the conventional treatment group will require an average of 96 ± 12 h of respiratory support based on previous departmental data. It is expected that integrating ultrasound diagnosis will promptly improve respiratory function, reducing the mechanical ventilation duration to an average of 86 ± 12 h. To compare the difference in mechanical ventilation duration between the groups, a two-sample independent *t*-test will be employed, setting α at 0.05 and ensuring a power of 80%. With a 1:1 ratio between the groups, sample size estimation using PASS15 indicates that each group will require 24 patients, totaling 48 patients. Considering the potential risk of underestimating sample size due to low effect size and dropout rates, the actual sample size will be 60 patients (48 ÷ 80% = 60), with 30 patients in each group.

### 2.13 Statistical analysis

The demographic and clinical characteristics of both the ultrasound and control groups will be described using means and standard deviations, medians and ranges, or ratios and percentages. Statistical analyses will be conducted using SPSS 20.0 software. Normally distributed continuous data will be presented as means ± standard deviations, with inter-group comparisons assessed using independent two-sample *t*-tests. Non-normally distributed continuous data will be presented as M (Q1, Q3), with inter-group comparisons analyzed using the Mann-Whitney *U*-test. Categorical data will be expressed as counts (%), and inter-group comparisons will be evaluated using the *χ*^2^ test, continuity-corrected *χ*^2^ test, or Fisher's exact test. A *P*-value of less than 0.05 will be considered statistically significant.

## 3 Discussion

In recent years, there has been an increasing focus on using LUS to predict the need for PS administration. De Martino et al. indicated the potential of LUS for predicting PS administration (11). Rodriguez-Fanjul et al. enrolled 56 very preterm infants, with a GA of less than 32 weeks, and divided them into a lung ultrasound and control group. In the lung ultrasound group, PS administration was guided by LUS or FiO_2_ levels within 1 h after birth, while in the control group, it was based on the FiO_2_ level alone. Ultimately, they discovered that the lung ultrasound group exhibited better oxygenation statuses and lower FiO_2_ requirements post-PS administration (23). Raimondi et al. categorized preterm infants with a GA of less than 34 weeks by age and conducted weekly lung ultrasonography and scoring from birth. They concluded that LUS showed a significant correlation with oxygenation status and could partly predict the occurrence of late-stage BPD (24). At present, there are also ongoing multi-center randomized controlled clinical trials (25).

Regarding the selection of the time point for the first pulmonary ultrasound after birth, previous studies have mostly set the time point between immediately and 3 h after birth (11, 26). As early as 2009, the “golden hour” management concept was introduced into the early treatment of premature infants and is believed to have improved the long-term prognosis (17, 18). In this study, the selection of the first pulmonary ultrasound examination at 1 h after birth was also based on this concept. Our NICU ward is relatively close to the delivery room, and only patients born in our hospital without severe asphyxia are selected as the study objects. Some of these patients can be transferred to the NICU ward within 15 min after birth and do reach a stable state within a short time after birth (owing to factors such as positioning of the vascular access, stabilization of ventilatory parameters, and thermal homeostasis) (18). Therefore, the subjects had received stable respiratory support for a long enough time at 1 h after birth for a pulmonary ultrasound assessment to be appropriate. The selection of LUS >8 as the cut-off value of PS in this study is also based on the results of previous studies (11, 23, 25, 27).

Although there are many clinical studies attempting to use LUS to evaluate the administration of PS, it has been suggested that LUS may fail to precisely evaluate the severity of lung lesions in patients (28). Moreover, conditions other than NRDS can also lead to elevated LUS, such as severe pulmonary edema, meconium aspiration syndrome, and pneumonia. Relying solely on LUS for determining the administration of PS is considered inadequate.

Based on prior research, this study protocol has been refined and improved in the following aspects: (1) Patient selection: very preterm infants with a GA of less than 32 weeks will be selected. According to previous study reports, the incidence of NRDS increases gradually with a decrease in GA. The incidence of NRDS in extremely preterm infants (GA ≤28 w) has been reported to be 93%; however, even among late preterm infants at 34 weeks GA, 10.5% of patients were diagnosed with NRDS (29, 30). In preterm infants below 32 weeks GA, early postnatal respiratory distress can be caused by a variety of causes. Differential diagnosis through traditional imaging is not only less sensitive but also brings about the challenges of radiation exposure. Patients often have delayed application or overuse of PS owing to misdiagnosis. Our study will include this population as the research object, hoping to identify NRDS more accurately and guide PS application through non-invasive methods in the early stage. (2) The clinical application of LUS was optimized: most previous studies judge whether to use PS only according to LUS, ignoring the possibility that other diseases causing respiratory distress may be misdiagnosed as NRDS. The severity of lung lesions caused by different diseases cannot be compared in parallel by LUS. In our study, the researchers first diagnosed NRDS through lung ultrasound and then assessed the severity of NRDS according to LUS and guided the application of PS. The combination of qualitative diagnosis and semi-quantitative assessment was more reasonable. In the diagnosis of NRDS by pulmonary ultrasound, the lesions may appear in any lung field; the pulmonary ultrasound guidelines clearly indicate that the lung lesions of NRDS mainly appear posteriorly, and therefore, the examination of the posterior region should not be ignored (19). In previous studies, in order to avoid moving patients, only the anterior and lateral lung fields were examined and scored, which may affect the accuracy of results (25, 31). In our study, according to the pulmonary ultrasound guidelines for lung partitioning, the anterior, lateral and posterior lungs were comprehensively evaluated and the scores of each lung field were recorded; the final total score was then calculated, which will be more accurate than the calculations in previous studies. (3) Timing of lung ultrasonography: previous studies have limited time points for lung ultrasound assessments, usually within the first 24 h post-birth. However, in the early postnatal period, premature infants experience rapid changes in their conditions and may require repeated administration of PS. Therefore, in our study design, the ultrasound group will undergo multiple lung ultrasound examinations within the first hour after birth, 4–10 h after the initial PS administration, prior to subsequent PS administration, and at 24- and 72-h post-birth. This will be the highest frequency of lung ultrasound examinations among known studies of the same type, and this approach allows for better monitoring of early postnatal lung ultrasound dynamics. Additionally, follow-up lung ultrasonography will be conducted at 1, 2, and 4 weeks after birth, as well as before discharge, thereby providing a basis for prognostic assessment; (4) Treatment method: following the 2022 European Consensus Guidelines on the Management of Respiratory Distress Syndrome, NCPAP combined with LISA was identified as the preferred treatment for preterm infants with spontaneous breathing after birth (2). Therefore, in this study, for infants receiving non-invasive ventilation (NCPAP or NIPPV) and exhibiting spontaneous breathing, priority will be given to the LISA method to reduce the risk of airway injury. This study aims to combine non-invasive diagnosis methods with non-invasive treatment to reduce the requirement for early invasive operations after the birth of preterm infants.

This study has several potential limitations: (1) Blinding ultrasound physicians and neonatologists to the grouping situation is impractical because the decision on PS administration relies on lung ultrasound results post-patient enrollment. However, subsequent ultrasound results, except for the first one, will not be disclosed to the clinicians to avoid influencing decisions on subsequent treatment. (2) Premature infants with respiratory distress will not be immediately grouped after birth but will instead be grouped upon arrival at the NICU. Nevertheless, it is stipulated that the same T-piece resuscitator should be used for non-invasive respiratory support during resuscitation in the delivery room and transportation process, and the infants should arrive at the NICU within 15 min after birth. (3) This study has a small sample size and is conducted at a single center. Future research will need to expand the study scope and increase the sample size.

## 4 Conclusion

If the study hypotheses are confirmed, it may establish a foundation for early PS administration in very premature infants based on NRDS diagnosis and LUS in the early postnatal period. This approach aims to optimize PS utilization, minimize x-ray exposure, reduce mechanical ventilation duration, lower BPD incidence, and ultimately enhance patient prognosis.
